# *Prevotella*-to-*Bacteroides* ratio predicts body weight and fat loss success on 24-week diets varying in macronutrient composition and dietary fiber: results from a post-hoc analysis

**DOI:** 10.1038/s41366-018-0093-2

**Published:** 2018-05-17

**Authors:** Mads F. Hjorth, Trine Blædel, Line Q. Bendtsen, Janne K. Lorenzen, Jacob B. Holm, Pia Kiilerich, Henrik M. Roager, Karsten Kristiansen, Lesli H. Larsen, Arne Astrup

**Affiliations:** 1 Department of Nutrition, Exercise and Sports, University of Copenhagen, Frederiksberg, Denmark; 2 Laboratory of Genomics and Molecular Biomedicine, Department of Biology, University of Copenhagen, Copenhagen, Denmark; 3 National Food Institute, Technical University of Denmark, Kgs. Lyngby, Denmark; 4 Institute of Metagenomics, BGI-Research, Shenzhen, 518083 China

**Keywords:** Randomized controlled trials, Obesity

## Abstract

**Background/objectives:**

Individuals with high pre-treatment bacterial *Prevotella*-to-*Bacteroides* (*P/B*) ratio have been reported to lose more body weight on diets high in fiber than subjects with a low *P/B* ratio. Therefore, the aim of the present study was to examine potential differences in dietary weight loss responses between participants with low and high *P/B*.

**Subjects/methods:**

Eighty overweight participants were randomized (52 completed) to a 500 kcal/d energy deficit diet with a macronutrient composition of 30 energy percentage (E%) fat, 52 E% carbohydrate and 18 E% protein either high (≈1500 mg calcium/day) or low ( ≤ 600 mg calcium/day) in dairy products for 24 weeks. Body weight, body fat, and dietary intake (by 7-day dietary records) were determined. Individuals were dichotomized according to their pre-treatment *P/B* ratio derived from 16S rRNA gene sequencing of collected fecal samples to test the potential modification of dietary effects using linear mixed models.

**Results:**

Independent of the randomized diets, individuals with high *P/B* lost 3.8 kg (95%CI, 1.8,5.8; *P* < 0.001) more body weight and 3.8 kg (95% CI, 1.1, 6.5; *P* = 0.005) more body fat compared to individuals with low *P/B*. After adjustment for multiple covariates, individuals with high *P/B* ratio lost 8.3 kg (95% CI, 5.8;10.9, *P* < 0.001) more body weight when consuming above compared to below 30 g fiber/10MJ whereas this weight loss was 3.2 kg (95% CI, 0.8;5.5, *P* = 0.008) among individuals with low *P/B* ratio [Mean difference: 5.1 kg (95% CI, 1.7;8.6, *P* = 0.003)]. Partial correlation coefficients between fiber intake and weight change was 0.90 (*P* < 0.001) among individuals with high *P/B* ratio and 0.25 (*P* = 0.29) among individuals with low *P/B* ratio.

**Conclusions:**

Individuals with high *P/B* lost more body weight and body fat compared to individuals with low *P/B*, confirming that individuals with a high *P/B* are more susceptible to weight loss on a diet rich in fiber.

## Introduction

Current interventions and policies have failed to stop the rise in the global obesity epidemic. Numerous randomized controlled trials have compared a myriad of diets for the treatment of obesity based on the assumption that one diet fits all without being able to provide strong evidence in favor of one or the other [1–5].

Accumulating evidence is linking gut microbiota to obesity. Overall, individuals with obesity show decreased bacterial diversity [6] and gene richness [7, 8] and fecal transplantation even suggest a causal relationship between the microbiome and obesity [9–11]. The composition of the gut microbiota has the potential to affect the efficacy of energy harvest [12] particularly though the fiber-utilization capacity [13], to influence the secretion of gastrointestinal hormones affecting appetite [14, 15], and potentially to affect human behavior through the gut-brain-axis [16]. Of note, the metabolic responses to different diets were recently shown to vary between individuals depending on the composition of their gut microbiota [17, 18]. Therefore, the human gut microbiota has the potential to play a pivotal role in obesity management through personalized nutrition.

Studies have suggested that the microbiota of individuals can be clustered into so-called enterotypes based on the genus composition [19] suggesting that such compositional differences may reflect dietary intake and determine the individual responses to different diets. The *Bacteroides*-driven enterotype is reported to be predominant in individuals with a high intake of protein and animal fat (Western diet), whereas the *Prevotella-*driven enterotype appears predominant in individuals that consume diets rich in carbohydrate and fiber [20–22]. The intestinal microbial communities are resilient and difficult to change through dietary interventions [20, 21, 23, 24], unless extreme changes, such as complete removal of carbohydrates from the diet, are introduced [25]. However, only a limited number of studies have related microbial enterotypes to health markers, such as cholesterol and LDL [14, 22–24]. In a randomized clinical study we recently reported that participants with high *Prevotella*-to-*Bacteriodes* (*P/B)* ratio were more susceptible to lose body fat on diets high in fiber than subjects with a low *P/B* ratio [24]. Furthermore, participants with no detectable *Prevotella* spp. had a weight loss response similar to that of participants with high *P/B* ratio, suggesting that other bacterial genera might also be involved.

The aim of the present study was to validate this recent finding [24] by re-analyzing an independent 24-week dietary intervention study [26] for potential differences in weight loss response between participants with no detectable *Prevotella* spp., low *P/B* ratio, and high *P/B* ratio independently of the allocated diets and stratified by macronutrient and fiber intake from the 7-day dietary records. As previously reported [26] no difference in macronutrient composition, dietary fiber, or 24 week weight loss response was observed between the two allocated diets (high and low diary). Therefore, it was hypothesized that participants stratified into the low and high *P/B* ratio group would not respond differently to the two allocated diets. However, as both the allocated diets were relatively low in fat and high in protein, carbohydrate and dietary fiber, it was hypothesized that participants with high *P/B* ratio (and possibly also participants with no detectable *Prevotella* spp.) would lose more body weight and body fat compared to participants with low *P/B* ratio, especially when consuming a diet high in dietary fiber evaluated by 7-day dietary records.

## Materials and methods

As previously reported [26], potential participants were invited for an information meeting and a physical examination at a screening visit after signing the informed consent. Inclusion criteria were: (1) Habitual calcium intake < 800 mg/d, (2) No dairy food allergies, (3) No infectious or metabolic diseases, (4) No use of dietary supplements during the study or 6 months prior to the study, (5) No use of cholesterol lowering medicine or other medication that would be expected to affect the study outcomes, (6) No gastrointestinal diseases, (7) No participation in other clinical studies, and (8) Women could not be pregnant or lactating. A total of 96 overweight or obese (BMI 28–36 kg/m^2^) men and women aged 18-60 years met the inclusion criteria of whom 80 participants were included in the study, which 52 completed all 24 weeks. In this randomized, controlled, parallel design, participants were allocated to a 500 kcal (2100 kJ)/d energy deficit diet with a macronutrient composition of 30 energy percentage (E%) fat, 52 E% carbohydrate and 18 E% protein that was either high (≈1500 mg calcium/day of which 1200 mg calcium/day should be consumed in the form of dairy products) or low (≤600 mg calcium/day) in dairy products during a 24 week period. Energy requirements were determined at the dietary counselling visit at baseline and adjusted after 12 weeks based on body weight, gender, age [27], and physical activity level assessed by Baeckes questionnaire [28]. Randomization was performed by staff not involved in screening of the participants and performed according to four strata: (1) women with BMI ≤ 31 kg/m^2^, (2) women with BMI > 31 kg/m^2^, (3) men with BMI ≤ 31 kg/m^2^, (4) men with BMI > 31 kg/m^2^. The participants attended seven individual dietary counselling visits and one group session scheduled at week 0, 2, 4, 8, 12, 16, 20, and 24 where body weight was also recorded to the nearest 0.1 kg (Lindeltronic 8000 S, Lindell’s, Malmo, Sweden). At baseline and after 24 weeks, a fecal sample was collected at home, immediately cooled, transported to the Department as soon as possible, and aliquots were stored immediately at −80°C. Bacterial DNA was extracted from frozen fecal samples using the NucleoSpin® soil kit (Macherey-Nagel, Düren, Germany), 5 ng DNA was used to amplify the V3 + V4 region of 16S rDNA genes, and operational taxonomic unit (OTU) picking was performed with 97% sequence similarity as previously described [26]. The relative abundances of sequences assigned to the *Prevotella* and *Bacteroides* genera were summarized. Furthermore, fasting blood samples were drawn at baseline, from where the concentrations of plasma glucose and serum insulin were analyzed as described elsewhere [26]. At baseline and week 24, body composition was determined by DXA (Lunar Prodigy DXA, Madison, USA) during standardized conditions. Finally, 7-day dietary records were obtained at both week 12 and 24, of which the mean value was calculated. From these mean values the intake of carbohydrate, protein, fat, and dietary fiber were categorized as being low or high based on the median split. Participants were instructed not to alter their habitual lifestyle throughout the study period beyond the instructions regarding the intervention and furthermore to refrain from physical activity, medicine and alcohol 48 h prior to the visits. More information about the study can be found elsewhere [26].

The study was conducted according to the guidelines laid down in the Declaration of Helsinki and all procedures involving human subjects were approved by the Danish National Committee on Health Research Ethics. Written informed consent was obtained from the participants after receiving oral and written information about study procedures. The study was registered on clinicaltrials.gov with the identifier: NCT01199835.

### Statistics

Two pre-treatment *P/B* groups were identified by plotting, for each sample, the log-transformed-relative abundance of *Bacteroides* spp. versus the log-transformed-relative abundance of *Prevotella* spp. as well as creating a histogram plotting frequency of the log-transformed-relative abundance of *Prevotella* spp./*Bacteroides* spp. As indicated by a recent study [24], subjects with no detectable *Prevotella* bacteria constituted a third group (named 0-*Prevotella*).

Baseline characteristics were summarized as mean ± standard deviation, median (interquartile range) or proportions (%). Differences between the three *P/B* groups were tested using one-way ANOVA (some variables transformed before analysis) with Bonferroni post-hoc test or Pearson’s chi-squared test.

Correlations between mean carbohydrate, fat, protein and fiber intake during the 24 weeks were analyzed by means of Pearson’s correlation coefficients and partial correlation coefficients (mutual adjustment of dietary components).

Differences in body weight change from baseline between *P/B* groups on the two allocated diets were analyzed by means of linear mixed models using all available measurements. The linear mixed models included the three-way interaction between diet x time x *P/B* group strata as well as all nested two-way interactions and main effects and comprised additional fixed effects including age, gender, baseline BMI, baseline fasting glucose and insulin as well as random effects for subjects. Secondly, a similar analysis was carried out only removing the allocated diet from the interaction term and instead including it as a covariate (same analysis was done for body fat as outcome). Finally, a similar analysis was carried out but only replacing the two allocated diets with median split of self-reported dietary intake (fat E%, protein E%, carbohydrate E%, and fiber g/10 MJ) one at a time (Model 2) while including the allocated diet as a covariate. Model 3 additionally include fat, protein, carbohydrate, and fiber as continues variables (except when included as exposure). Model 1 included no covariates.

Results are shown as correlations and mean weight change from baseline with 95% confidence interval (CI), and differences in weight change from baseline to end of study (week 24) were compared between allocated diets as well as median split of self-reported diets within each *P/B* group and between *P/B* groups (irrespective of diets) through pairwise comparisons using post hoc t-tests. All data were checked for normality and variance homogeneity. The level of significance was set at *P* < 0.05 and statistical analyses were conducted using STATA/SE 14.1 (Houston, USA).

## Results

Median (IQR) dietary distribution during the 24-weeks was 45.9 (43.6; 47.7) E% carbohydrates, 31.7 (29.3; 34.7) E% fats, 20.0 (18.1; 22.7) E% proteins, and 30.8 (26.1; 36.0) g/10 MJ dietary fibers.

**Fig. 1:**
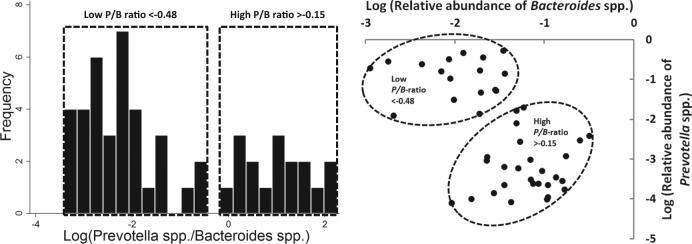
Identifying three distinct *Prevotella-to-Bacteriodes* groups Before the intervention participants were observed to form two distinct groups based on the log-transformed relative abundance of *Bacteroides* spp. and the log-transformed relative abundance of *Prevotella* spp. indicated with dotted lines and referred to as low (*n* = 27) and high (*n* = 17) *Prevotella-to-Bacteriodes* (*P/B*) groups. Participants with no detectable *Prevotella* spp., referred to as the 0-*Prevotella* group, constitute the third group (*n* = 8) but was excluded from this figure

The low and high *P/B* groups are indicated with dotted lines in Figure 1. A third group (*n* = 8) had no detectable *Prevotella* spp. and constitute a third group named 0-*Prevotella*.

Overall, body weight, BMI, and the relative abundance of *Bacteroides* spp. and *Prevotella* spp. differed between the three *P/B* groups (*P* ≤ 0.017), with the high *P/B* group having higher body weight, BMI, relative abundance of *Prevotella* spp. and lower relative abundance of *Bacteroides* spp. compared to the low *P/B* group (*P* < 0.05) (Table 1).

**Table 1:** Baseline characteristics of the study participants stratified into three groups according to *Prevotella*-to-*Bacteroides* (*P/B*) ratio

	0-*Prevotella*^1^ (*n* = 8)	Low *P/B* group (*n* = 27)	High *P/B* group (*n* = 17)	*P*-value
Age (year)	47.9 ± 6.8	43.4 ± 8.7	41.8 ± 11.5	0.33
Gender (% female/male)	100/0	88.9/11.1	76.5/23.5	0.24
Body weight (kg)	82.6 ± 4.6^a^	84.5 ± 11.4^a^	95.1 ± 12.0^b^	0.005
Body mass index (kg/m^2^)	30.7 ± 1.1^a, b^	29.7 ± 2.2^a^	31.9 ± 2.8^b^	0.017
Body fat (%)	48.7 ± 3.9	44.9 ± 4.1	44.4 ± 5.0^2^	0.069
Fasting glucose (mmol/L)	5.42 ± 0.46	5.55 ± 0.37	5.70 ± 0.55	0.33
Fasting insulin (pmol/L)	63.4 (47.0; 88.1)	38.5 (23.7; 69.3)^3^	47.8 (28.8; 54.6)	0.17
*Prevotella* (relative abundance)	0 (0; 0)^a^	0.0003 (0.0002; 0.001)^b^	0.155 (0.052; 0.278)^c^	<0.001
*Bacteroides* (relative abundance)	0.097 (0.032; 0.139)^a^	0.071 (0.036; 0.111)^a^	0.012 (0.007; 0.021)^b^	<0.001
*Prevotella*-to-*Bacteroides* ratio	–	0.004 (0.001; 0.012)	11.67 (3.11; 36.03)	

Data are presented as mean ± standard deviation, median (interquartile range) or proportions (%) and differences between the three *P/B* groups were tested using one-way ANOVA with Bonferroni post-hoc tests (some variables transformed before analysis) or Pearson’s chi-squared test. Different alphabets within a row (a, b, c) indicate significant differences (*P* < 0.05)

*P/B* *Prevotella*-to-*Bacteroides*

^1^0-*Prevotella* refers to the group of individuals with no detectable *Prevotella* spp. before intervention.

^2^*n* = 16 (missing data for one individual)

^3^*n* = 26 (missing data for one individual)

**Fig. 2:**
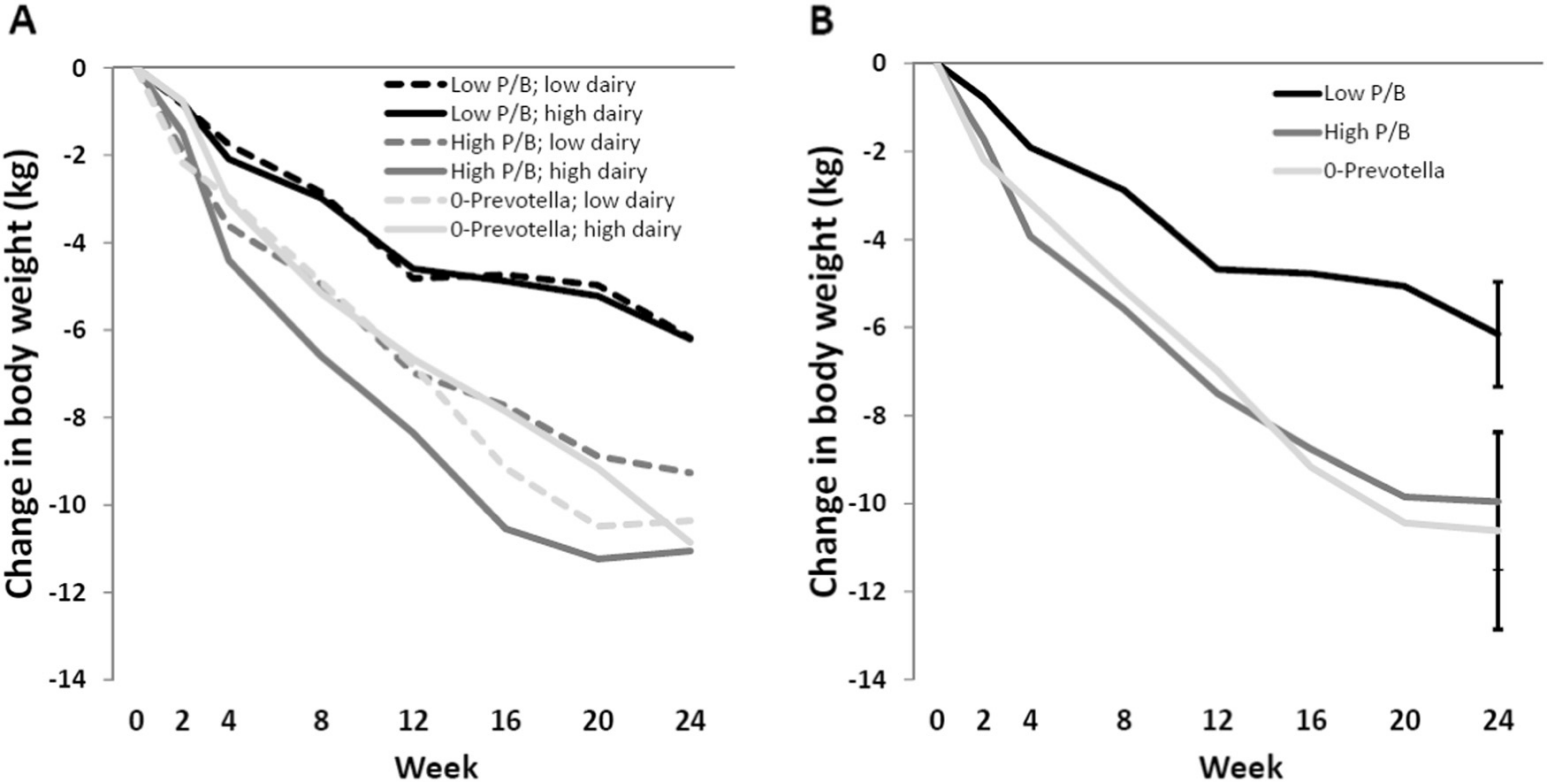
Change in body weight (**a**) between and (**b**) within diets when stratified into three groups according to *Prevotella*-to-*Bacteroides (P/B)* ratio. Data are presented as estimated mean weight change from baseline for each combination of the (**a**) diet-time-*P/B* strata interaction or (**b**) time-*P/B* strata interaction in the linear mixed models, which were additionally adjusted for age, gender, baseline BMI, fasting glucose, fasting insulin, (also diet allocation in panel **b**), and subjects. Differences in weight change from baseline were compared after 24 weeks through pairwise comparisons using post hoc *t*-tests and presented as mean weight change from baseline with 95% confidence intervals. No difference in weight change was observed between the two diets (low and high dairy) within any of the three *P/B* groups (all *P* ≤ 0.23) (see panel **a**). For clarity, confidence intervals were omitted from panel **a**. The two different diets were collapsed and differences in weight change between the three *P/B* groups were compared after 24 weeks (see panel b) § indicate significant difference between the low *P/B* group and each of the high *P/B* and 0-*Prevotella* group (both *P* < 0.001)

After the 24-week caloric restricted diet, no difference in 24 week body weight change was observed between the two allocated diets within the 0-*Prevotella* group [0.50 kg (95% CI, −5.84, 6.83; *P* = 0.88)], low *P/B* group [0.03 kg (95% CI, −2.28, 2.34; *P* = 0.98)], or high *P/B* group [1.79 kg (95% CI, −1.12, 4.70; *P* = 0.23)] (Fig. 2a).

Irrespective of the allocated diets, participants with a low *P/B* ratio lost 3.80 kg (95% CI, 1.77, 5.84; *P* < 0.001) and 4.47 kg (95% CI, 1.90, 7.04; *P* < 0.001) less body weight compared to participants with high *P/B* ratio and 0-*Prevotella*, respectively. No difference was observed between participants with high *P/B* ratio and 0-*Prevotella* [0.66 kg (95% CI, −2.16, 3.49; *P* = 0.65)] (Fig. 2b; Table 2). Likewise, participants with a low *P/B* ratio lost 3.80 kg (95% CI, 1.13, 6.48; *P* = 0.005) and 3.41 kg (95% CI, 0.11, 6.71; *P* = 0.043) less body fat compared to participants with high *P/B* ratio and 0-*Prevotella*, respectively. There was no difference in fat loss between participants with high *P/B* ratio and 0-*Prevotella* [0.40 kg (95% CI, −3.35, 4.14; *P* = 0.84)] (Table 2).

**Table 2:** Changes in body weight and body fat after 24 weeks when stratified into three groups according to *Prevotella*-to-*Bacteroides* ratio (*n* = 51)

	0-*Prevotella* (*n* = 8)	Low *P/B* (*n* = 26)	High *P/B* (*n* = 17)
∆Body weight (kg)	−10.62 (−12.86; −8.38)^a^	−6.15 (−7.34; −4.96)^b^	−9.96 (−11.50; −8.41)^a^
∆Body fat (kg)	−8.58 (−12.29; −4.87)^a^	−5.18 (−6.71; −3.66)^b^	−8.98 (−11.03; −6.95)^1a^

Data are presented as estimated mean body weight and body fat change from baseline and 95% confidence intervals for three *Prevotella-to-Bacteroides* groups after 24 weeks in the linear mixed models, which were additionally adjusted for age, gender, baseline BMI, fasting glucose, fasting insulin, diet allocation and random effects for subjects (only when analyzing body weight)

*P/B* *Prevotella*-to-*Bacteroides*

Different alphabets within a row (a, b) indicate significant differences (*P* < 0.05)

^1^*n* = 16 (missing data for one individual)

Macronutrient and fiber intake from the self-reported dietary intake during the 24 week was correlated as seen in Table S1.

In the fully adjusted model, participants with low *P/B* ratio lost more body weight when consuming a diet above the median in carbohydrate (%) and dietary fiber (g/10 MJ) (both *P* ≤ 0.008) whereas the high *P/B* ratio lost more body weight when consuming a diet above the median in carbohydrate (%), dietary fiber (g/10 MJ), and protein (%) (all *P* < 0.001) (Table 3) [Mean difference: Fat: 4.0 kg (0.6; 7.3, *P* = 0.02); Carbohydrate: 4.3 kg (1.3; 7.2, *P* = 0.004); Protein: 6.6 kg (3.0; 10.3, *P* < 0.001); Dietary fiber: 5.1 (1.7; 8.6, *P* = 0.003)]. Furthermore, participants in the 0-*Prevotella* group lost more body weight when consuming a diet above the median in carbohydrate (%) and fat (%) (both *P* ≤ 0.001).

**Table 3:** Change in body weight among the three *Prevotella*-to-*Bacteriodes* (*P/B*) groups stratified by median of self-reported dietary intake (*n* = 51)

		0-*Prevotella* (*n* = 8)		Low *P/B* (*n* = 26)		High *P/B* (*n* = 17)	
		Lower median	Higher median	Lower median	Higher median	Lower median	Higher median
Fat (E%)^1^		(*n* = 3)	(*n* = 5)	(*n* = 11)	(*n* = 15)	(*n* = 7)	(*n* = 10)
	M1	−6.3 (−9.8; −2.8)	−13.0 (−15.7; −10.3)^2^	−6.4 (−8.2; −4.6)	−5.6 (−7.2; −4.1)	−12.2 (−14.4; −9.9)	−7.5 (−9.4; −5.6)^2^
	M2	−6.0 (−9.5; −2.4)	−13.0 (−15.5; −10.5)^2^	−6.0 (−7.7; −4.3)	−5.9 (−7.5; −4.4)	−12.8 (−15.1; −10.6)	−8.1 (−10.0; −6.3)^2^
	M3	−3.0 (−6.2; 0.3)	−13.8 (−16.1; −11.6)^2^	−4.9 (−6.5; −3.4)	−6.9 (−8.3; −5.5)	−11.6 (−13.7; −9.4)	−9.6 (−11.3; −7.8)
Protein (E%)^1^		(*n* = 4)	(*n* = 4)	(*n* = 11)	(*n* = 15)	(*n* = 12)	(*n* = 5)
	M1	−11.1 (−14.1; −8.2)	−9.9 (−12.8; −6.9)	−6.5 (−8.3; −4.7)	−5.6 (−7.1; −4.1)	−7.6 (−9.3; −5.9)	−13.6 (−16.3; −11.0)^2^
	M2	−10.8 (−13.7; −8.0)	−8.9 (−11.8; −6.0)	−6.1 (−7.7; −4.5)	−5.9 (−7.4; −4.3)	−8.4 (−10.1; −6.8)	−14.8 (−17.6; −12.0)^2^
	M3	−10.4 (−13.1; −7.6)	−9.3 (−12.1; −6.4)	−6.1 (−7.8; −4.5)	−5.7 (−7.2; −4.2)	−8.6 (−10.3; −7.0)	−14.8 (−17.5; −12.1)^2^
Carbohydrate (E%)^1^		(*n* = 6)	(*n* = 2)	(*n* = 12)	(*n* = 14)	(*n* = 8)	(*n* = 9)
	M1	−9.2 (−11.5; −6.9)	−14.4 (−18.3; −10.4)^2^	−5.2 (−6.8; −3.6)	−6.6 (−8.1; −5.1)	−5.7 (−7.7; −3.7)	−12.7 (−14.5; −10.8)^2^
	M2	−9.1 (−11.3; −6.9)	−15.0 (−18.7; −11.3)^2^	−5.4 (−7.0; −3.8)	−6.4 (−7.9; −5.0)	−6.4 (−8.4;−4.4)	−13.1 (−14.9;−11.3)^2^
	M3	−8.3 (−10.3; −6.3)	−14.8 (−18.1; −11.4)^2^	−4.2 (−5.7; −2.8)	−7.7 (−9.1; −6.3)^2^	−6.0 (−8.0; −4.1)	−13.8 (−15.4; −12.1)^2^
Dietary fiber (g/10 MJ)^1^		(*n* = 2)	(*n* = 6)	(*n* = 12)	(*n* = 14)	(*n* = 8)	(*n* = 9)
	M1	−10.8 (−14.5; −7.0)	−10.4 (−12.6; −8.2)	−4.4 (−5.9; −2.8)	−7.3 (−8.8; −5.9)^2^	−4.7 (−6.6; −2.9)	−13.5 (−15.3; −11.8)^2^
	M2	−9.8 (−13.3; −6.2)	−11.1 (−13.2; −8.9)	−4.1 (−5.7; −2.5)	−7.2 (−8.5; −5.9)^2^	−5.7 (−7.5; −3.8)	−13.9 (−15.6; −12.3)^2^
	M3	−10.0 (−13.7; −6.3)	−11.0 (−13.2; −8.8)	−4.1 (−5.8; −2.4)	−7.3 (−8.7; −5.9)^2^	−5.6 (−7.6; −3.6)	−13.9 (−15.6; −12.2)^2^

Data are presented as estimated mean weight change from baseline and 95% confidence intervals for each combination of the dietary intake-time-*P/B* strata interaction after 24 weeks in the linear mixed models, which were adjusted for subject as random effects (M1). In model 2 (M2) additional adjustments for age, gender, baseline BMI, fasting glucose, fasting insulin, and diet allocation as fixed factors were performed. Model 3 (M3) include M2 + additional adjustments of fat E%, protein E%, carbohydrate E%, and fiber g/10MJ as continues variables (except when included as exposure). The displayed *n* is from M1 at week 24

*P/B* Prevotella-to-Bacteroides

^1^The approximate median value among the 49 participants having self-reported dietary intake was used as cut-off and was as following: Fat (31 E%), Protein (20 E%), Carbohydrate (46 E%), Fiber (30 g/10 MJ)

^2^Significant different (*P* < 0.05) within *P/B*-group between lower and higher median of dietary component

**Fig. 3:**
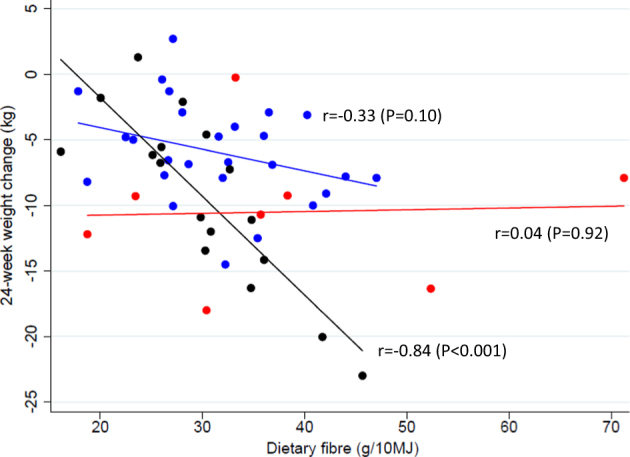
Scatter plots between dietary fiber and 24-week weight loss stratified by three *Prevotella/Bacteroides* groups. Blue: Low *P/B*-ratio (*n* = 26); Black: High *P/B*-ratio (*n* = 17); Red: 0-*prevotella* (*n* = 8). Pearson’s correlation coefficients are presented on the figure

Among individuals with high *P/B* ratio, fat (%) (*r* = 0.59), protein (%) (*r* = −0.58), and fiber (g/10 MJ) (*r* = −0.84) were significantly correlated with 24-week weight change (*P* ≤ 0.015) (Fig. 3 & Figure S1), but only fiber intake remained significant after adjusting for multiple covariates (*r* = 0.90, *P* < 0.001) (Table S2).No significant correlations was found between dietary components and weight loss among subjects with low P/B or 0-*Prevotella*.

The correlation coefficient between baseline and post-intervention log(*P/B*-ratio) was 0.87 (*P* < 0.001) as illustrated in Figure S2 emphasizing that the *P/B*-ratio overall remained stable during 24 weeks despite the observed weight loss.

## Discussion

As hypothesized, participants with no detectable *Prevotella* spp. and high *P/B* ratio lost approximately 4 kg more during 24 weeks compared to participants with low *P/B* ratio. Furthermore, this increased weight loss response among participants with no detectable *Prevotella* spp. and high *P/B* ratio was associated with individual macronutrient composition and dietary fiber intake estimated from 7-day dietary records. Specifically, among participants with high *P/B* ratio fiber intake above the median resulted in weight loss being more than twice as large, thereby explaining the entire weight loss difference between the low and high *P/B* groups. Finally, no differences in weight loss between the two allocated diets, differing in calcium, were observed for any of our three *P/B* groups. The present study serves as a validation of our recent observation showing an interaction between *P/B* ratio and dietary intake on weight and fat loss response in a dietary intervention studies [24].

Recently, the distinction of enterotypes as discrete clusters was challenged by studies suggesting that enterotype distribution is continuous and that information may be masked within these enterotype clusters [29, 30]. The three *P/B* groups in the present study were not as discrete as in our previous study [24]; however, the population could be divided with only few individuals possibly being intermediate. Furthermore, a comparison of the pre –and post interventional *P/B*-ratio shows good correlation and classification agreement, emphasizing that these ratios are very stable as previously reported [23]. From these results we cannot conclude if the *P/B* ratio is causally related to the different effects of the diets or simply a marker of something else that we did not measure. However, the study highlights the relative abundance of *Prevotella* spp. as important in the classification of microbiota profiles. In agreement herewith, we recently observed that subjects with no detectable *Prevotella* spp. responded differently than subjects in the low *P/B* group following a dietary intervention [24]. Although these findings were confirmed here, as the 0-*Prevotella* group lost more body weight compared to the low *P/B* group and supposedly lost more weight when consuming diets higher in carbohydrates and/or fat, this 0-*Prevotella* group only consisted of 8 participants. Therefore, these observations need further investigations to make solid conclusions.

Administration of short chain fatty acids (SCFA) have been reported to result in a wide range of health benefits including improvements in blood lipid profiles, glucose homeostasis, body composition, and reduced body weight [31]. However, studies tend to investigate all SCFA as a whole and neglect to report the specific effects associated with the individual SCFA with the most abundant being acetate, propionate, and butyrate [31]. Members of the phylum *Bacteroidetes* are known to be efficient degraders of dietary fiber and include the genera *Bacteroides and Prevotella* [32]. *In vitro* the *Prevotella*-driven and *Bacteriodes*-driven microbiota have been shown to produce different amounts and profiles of SCFA from the same carbohydrate substrates [13]. Therefore, the differences in *P/B* ratio in the present study, observed to affect the weight loss responsiveness to a fiber rich diet, could potentially be explained by the efficacy of energy harvest primarily as SCFA [12] or that the production of SCFA affects appetite either directly in the brain or through different signaling pathway influencing the secretion of gastrointestinal hormones [15, 31]. Improvements in post-prandial blood glucose and insulin after dietary fiber intake were recently found to be positively associated with the abundance of *Prevotella* [33]. Therefore, the importance of pre-treatment fasting glucose and insulin to determine the optimal diet for weight management [34–36], might also be linked to gut microbiota profiles, and we adjusted for fasting glucose and fasting insulin. However, independent of the mechanisms, the three *P/B* ratio groups may serve as a biomarker to predict future weight loss success on specific diets.

Limitations of the study include that the study was not designed to examine for differences in responsiveness according to *P/B* ratio, and it is a matter of chance that we had enough participants in each group to provide statistical power for analyses. However, the post-hoc approach can also be looked upon as a strength as the study was double-blinded with respect to the *P/B* ratio of the participants, and the identified difference in dietary responsiveness cannot have been influenced by knowledge of the participants or investigators. Furthermore, when stratifying on *P/B* ratio, the randomized study design that should balance out known and unknown confounders are weakened, which is why we adjusted for a number of baseline characteristics, including age, gender, and BMI. Although some of the analyses, especially those for individuals with 0-*Prevotella*, are based on relatively small numbers and the validity therefore could be questioned, these findings are consistent with our previous findings [24], suggesting robustness of our findings. On the other hand, the individuals in the present study with no detectable *Prevotella* bacteria at baseline primarily belonged to the low *P/B* group after the 24-week intervention (see Figure S2). Furthermore, the present results are partly based on self-reported dietary data during a controlled dietary intervention study with regular dietetic counseling of the participants. As the individual differences in macronutrient and dietary fiber consumption during the trial were found to influence weight loss responsiveness among the high *P/B* group, we speculate that free-living dietary intake, when not counseled by dieticians, would have an even bigger effect. In the current study, as well as our recently published study [24], we observed that individuals characterized with a high *P/B*-ratio tended to have a higher baseline BMI. However, as only the individuals with high *P/B*-ratio that consumed more fibers lost more weight, regression towards the mean is not likely to play a major role. Furthermore, baseline BMI was recently found to be identical whether dominated by *Prevotella* or *Bacteriodes* among >100 diabetic patients [37]. At present time, the main limitation when using the *P/B* ratio as a pre-treatment determinant of dietary weight loss among individual is the slightly deviating cut-offs compared to previously reported [24]. These differences in cut-off between studies could reflect population specific *P/B* ratios; however, more likely they reflect differences in the methodology of the bacterial profiling of *Prevotella* spp. and *Bacteroides* spp., where the present study applied 16S rRNA gene sequencing whereas the previous study applied quantitative polymerase chain reaction (qPCR) [23, 24]. Therefore, future use of the *P/B* ratio to determine individual dietary weight loss response on different diets would need a specific reference methodology or at least take the specific methodology used into consideration. It should furthermore be noted that the fecal microbiota primarily reflects the microbiota of the distal part of the colon. Therefore, it remains unknown how the fecal *P/B* ratio relates to the bacterial composition in the proximal part of the colon as well as the small intestine.

Finally, industrialized populations consuming a Western diet have microbiotas that are dominated by the family *Bacteroidaceae* (composed of four genera including *Bacteroides*) whereas traditional populations across Africa, Asia, and South America have microbiotas that are dominated by the family *Prevotellaceae* (composed of four genera including *Prevotella*) that has been found to fluctuate according to foods available during different seasons [38]. Although we know that it is difficult to change the *P/B* ratio though dietary interventions [20, 21, 23, 24], we know that short term diets without carbohydrates [25] and seasonal difference [38] affect these genera and thereby provide evidence that we might be able to manipulate the *P/B* ratio.

In summary, we successfully validated the pre-treatment *P/B* ratio to be an important biomarker associated with dietary weight loss. Specifically, we found that participants having high *P/B* ratio had a larger 24 week weight loss compared to participants with low *P/B* ratio when advised to eat a healthy energy restricted diet (carbohydrate: 52E%, fat: 30 E% and protein: 18E%). This ≈4 kg differences in weight loss between high and low *P/B* ratio groups was explained by interaction with the actual diet consumed. Thus, individuals with a high *P/B* ratio were more susceptible to body weight loss, compared to individuals with a low *P/B* ratio, specifically on a diet rich in fiber and possibly also high in carbohydrates, high in protein and low in fat.

## Electronic supplementary material

Table S1

Table S2

Supplementary figure legends

Figure S1

Figure S2
