# Supplementary material for: Prevotella-to-Bacteroides ratio predicts body weight and fat loss success on 24-week diets varying in macronutrient composition and dietary fiber: results from a post-hoc analysis
Source: Int J Obes (Lond). 2018 May 17;43(1):149–57. doi: 10.1038/s41366-018-0093-2 (PMC6331389; doi:10.1038/s41366-018-0093-2)
Supplement: Supplementary file 1 — Table S1 [file 41366_2018_93_MOESM1_ESM.docx]

| **Table S1**: Correlation and partial correlation coefficients between mean carbohydrate, fat, protein and fiber intake during the 24 weeks (n=51). | | | |
| --- | --- | --- | --- |
|  | Carbohydrate (%) | Fat (%) | Protein (%) |
| Fat (%) | -0.68**/ -0.85** |  |  |
| Protein (%) | -0.11/ -0.64** | -0.46**/ -0.71** |  |
| Fiber (g/10 MJ) | -0.05/ -0.33* | -0.31*/-0.37* | 0.32*/-0.06 |
| First number is Pearson’s correlation coefficients between two dietary components. Second number is the partial correlation coefficients between two dietary components (adjusting for the remaining two dietary components).  *P < 0.05, **P < 0.001. | | | |
