# Supplementary material for: Prevotella-to-Bacteroides ratio predicts body weight and fat loss success on 24-week diets varying in macronutrient composition and dietary fiber: results from a post-hoc analysis
Source: Int J Obes (Lond). 2018 May 17;43(1):149–57. doi: 10.1038/s41366-018-0093-2 (PMC6331389; doi:10.1038/s41366-018-0093-2)
Supplement: Supplementary file 2 — Table S2 [file 41366_2018_93_MOESM2_ESM.docx]

| **Table S2**: Correlation and partial correlation coefficients between 24-week weight change and each of mean carbohydrate, fat, protein and fiber intake during the 24 weeks (n=51). | | | | |
| --- | --- | --- | --- | --- |
|  | Carbohydrate (%) | Fat (%) | Protein (%) | Fiber (g/10 MJ) |
| All | -0.08/-0.06/-0.11 | 0.22/0.16/-0.07 | -0.19/-0.16/-0.12 | -0.37*/-0.37*/-0.32* |
| 0-*Prevotella* (n=8) | -0.12/-0.26/-0.50 | -0.21/-0.27/0.08 | 0.20/0.20/0.27 | 0.04/0.04/0.21 |
| Low *P/B* group (n=26) | 0.01/0.19/-0.02 | 0.11/-0.04/-0.09 | -0.10/-0.09/0.02 | -0.33/-0.29/-0.25 |
| High *P/B* group (n=17) | -0.27/-0.34/0.60 | 0.59*/0.52/0.57 | -0.58*/-0.54*/0.39 | -0.84**/-0.90**/-0.90** |
| First number is Pearson’s correlation coefficients between 24-week weight change and one dietary component. Second number is the partial correlation coefficients between 24-week weight change and one dietary component adjusted for age, gender, baseline BMI. Third number is additionally adjusting for the remaining three dietary components.  *P < 0.05, **P < 0.001. | | | | |
