# Supplementary material for: Prevotella-to-Bacteroides ratio predicts body weight and fat loss success on 24-week diets varying in macronutrient composition and dietary fiber: results from a post-hoc analysis
Source: Int J Obes (Lond). 2018 May 17;43(1):149–57. doi: 10.1038/s41366-018-0093-2 (PMC6331389; doi:10.1038/s41366-018-0093-2)
Supplement: Supplementary file 3 — Supplementary figure legends [file 41366_2018_93_MOESM3_ESM.docx]

**Figure S1: Scatter plots between dietary composition and 24-week weight loss stratified by three *Prevotella/Bacteroides* groups.**

Blue: Low *P/B*-ratio (n=26); Black: High *P/B*-ratio (n=17); Red: 0-*prevotella (n=8)*. Pearson’s correlation coefficients between energy intake and weight change were 0.01 (P=0.97), 0.32 (P=0.20), and 0.08 (P=0.86) among subjects with low *P/B*-ratio, high *P/B*-ratio and 0-*Prevotella*, respectively. The remaining correlation coefficients are listed in Table S2.

**Figure S2: Scatterplot between baseline and 24-week post-intervention log(*Prevotella/Bacteriodes)***

Lines (x=-0.25; y=-0.25) separate low and high *Prevotella/Bacteriodes*-ratio. Subjects with no detectable *Prevotella* have the value “-4” on the figure. Pearson’s correlation coefficient (not including subjects with 0-*Prevotella*) is 0.87 (P<0.001; n=42). Two microbiota samples are missing at 26 weeks (total n=50).
