# Supplementary material for: Prevotella-to-Bacteroides ratio predicts body weight and fat loss success on 24-week diets varying in macronutrient composition and dietary fiber: results from a post-hoc analysis
Source: Int J Obes (Lond). 2018 May 17;43(1):149–57. doi: 10.1038/s41366-018-0093-2 (PMC6331389; doi:10.1038/s41366-018-0093-2)
Supplement: Supplementary file 4 — Figure S1 [file 41366_2018_93_MOESM4_ESM.docx]

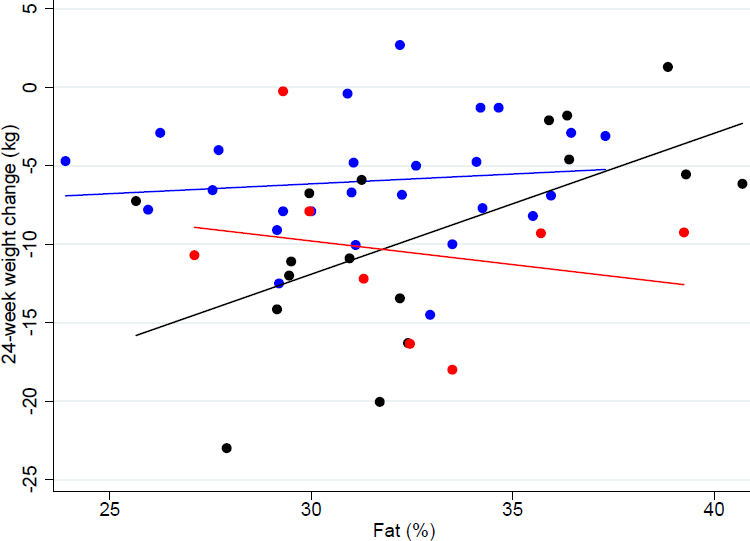

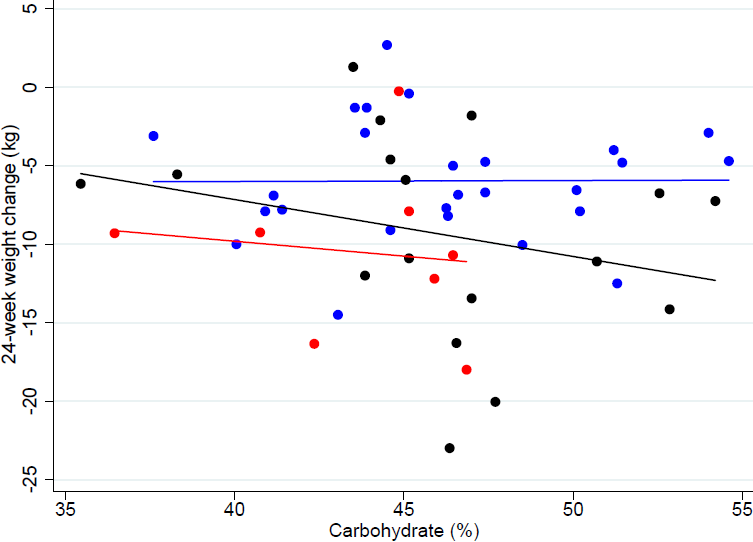


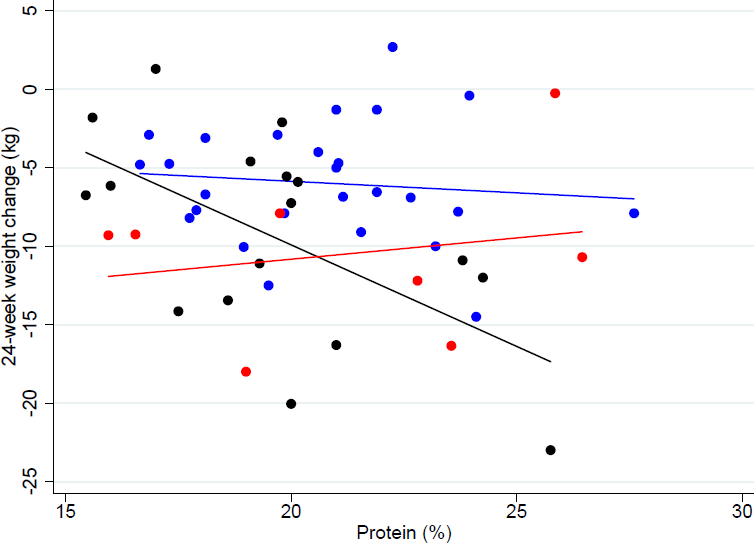

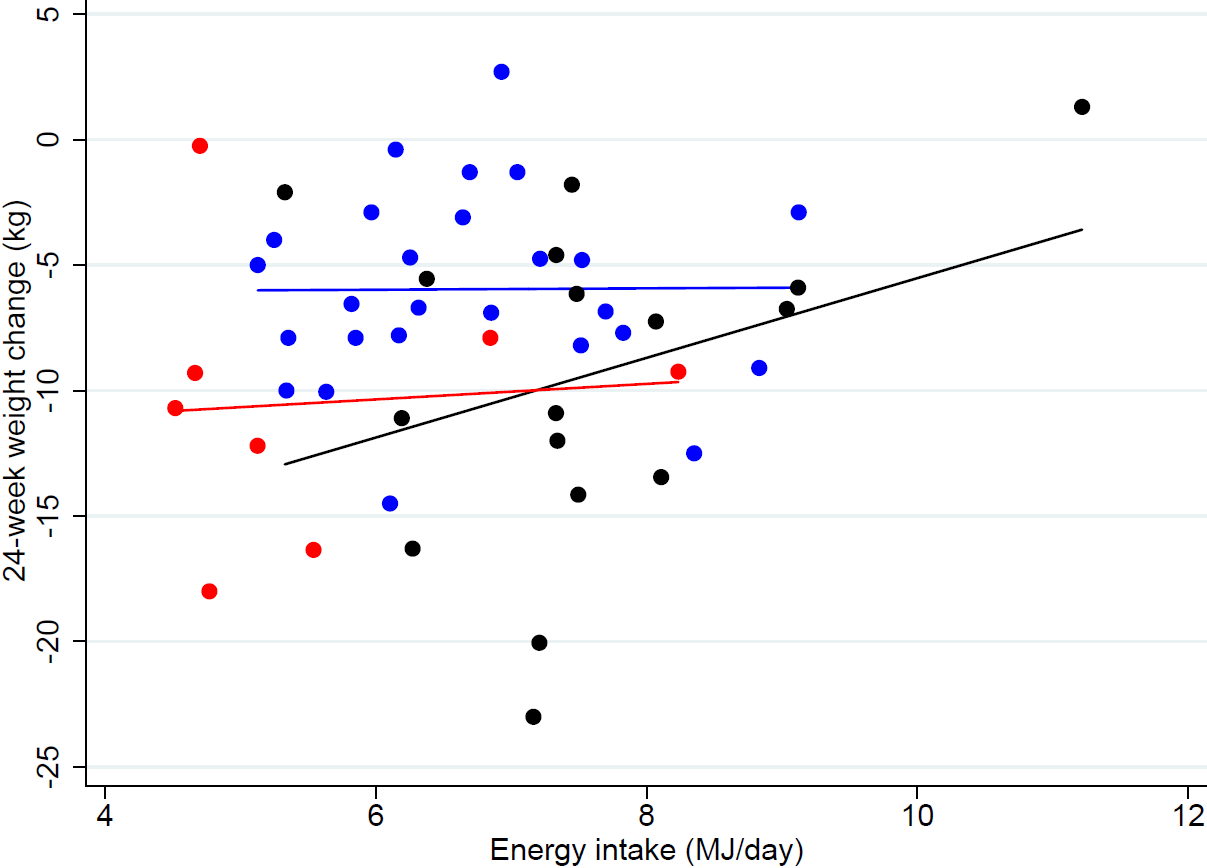


**Figure S1: Scatter plots between dietary composition and 24-week weight loss stratified by three *Prevotella/Bacteroides* groups**
